# Subtract to solve: a pilot study testing implicit and experiential interventions against additive bias

**DOI:** 10.3389/fcogn.2025.1624526

**Published:** 2025-10-02

**Authors:** Maria Adriana Neroni

**Affiliations:** Department of Education, Psychology and Communication Sciences, Suor Orsola Benincasa University, Naples, Italy

**Keywords:** additive bias, implicit association test (IAT), creative problem solving, reasoning, experiential learning

## Abstract

When seeking to transform an object, idea, or situation, individuals often default to adding new components rather than removing existing ones, a cognitive tendency known as additive bias. Although recently formalized in cognitive science, strategies to mitigate this bias remain limited. This pilot study investigated the potential of the additive bias Implicit Association Test (ad-IAT) as a scalable educational tool for raising awareness of additive bias and promoting subtractive thinking. Sixty participants were randomly assigned to one of three conditions: ad-IAT, experiential learning, or control. In Session 1, all participants completed a familiarization task with a digital grid, which served as the foundation for the subsequent tasks in the study. In Session 2, participants completed either the ad-IAT (with personalized feedback), a grid-based experiential task emphasizing subtractive efficiency or an unrelated gender IAT. In Session 3, all participants completed the same test grid, structured so that symmetry could be achieved more efficiently through subtraction than addition. Results showed that participants in the ad-IAT condition exhibited a strong implicit preference for additive concepts. Although differences in strategy use were not statistically significant across conditions, both the ad-IAT and experience groups demonstrated higher accuracy than the control group, with the experience group completing the task significantly faster. These findings suggest that both implicit and experiential interventions can reduce reliance on additive strategies, with the ad-IAT offering a time-efficient and scalable method for promoting metacognitive insight and behavioral change. Implications for creativity, education, and cognitive training are discussed.

## 1 Introduction

Additive bias refers to the cognitive tendency to solve problems by adding elements, even when subtraction would yield a more efficient or effective solution. Although this bias has long influenced human behavior and societal systems (Frederiks et al., 2015; Jannace and Camuffo, 2023; Kollmuss and Agyeman, 2002), it was formally conceptualized only recently in a landmark study by Adams et al. (2021). Across a series of experiments, Adams and colleagues consistently demonstrated that individuals default to additive rather than subtractive changes, even when subtraction is objectively preferable.

Far from being confined to specific domains, additive bias permeates many aspects of everyday life. It emerges in consumer behavior, such as the tendency to purchase and consume more than necessary, during sales seasons or at *all-you-can-eat* buffets, or to accumulate possessions rather than declutter (Kondo, 2014). In product design, it contributes to “feature creep,” the excessive accumulation of functions that ultimately undermines usability (Page, 2009; Thompson et al., 2005). On a broader scale, it fuels bureaucratic inefficiencies, overconsumption, and unsustainable practices (Frederiks et al., 2015).

Crucially, additive bias also hinders creativity. Creative thought often requires breaking away from habitual patterns and exploring counterintuitive possibilities (Finke et al., 1992; Crilly, 2015; Ward et al., 1999). According to the Geneplore model (Finke et al., 1992), creativity unfolds in two phases: a generative phase, in which multiple representations or pre-inventive structures are produced, and an exploratory phase, in which these are evaluated, refined, or transformed. Additive bias disrupts this cycle by narrowing the generative space, leading individuals to favor familiar additive solutions while overlooking simpler or more original alternatives.

Recognizing the limitations imposed by additive bias highlights the overlooked value of its counterpart: subtraction. Though often neglected, subtractive thinking holds significant untapped potential. Approaching problems with subtraction in mind can uncover alternative and sometimes superior solutions that might otherwise remain hidden. Examples of this mindset include balance bikes, which aid children's cycling skills by removing pedals (Klotz, 2021); Braess's paradox, which demonstrates that eliminating certain roads can paradoxically reduce traffic congestion (Braess et al., 2005); and minimalist design principles, exemplified by Google's uncluttered search interface and Apple's streamlined product aesthetics (Gallo, 2011). Collectively, these cases show that creativity and innovation can arise not only from accumulation but also from the deliberate reduction or removal of elements.

Additive bias is influenced not only by cognitive mechanisms but also by embodied, linguistic, and cultural forces. Research in numerical cognition shows a fundamental asymmetry: addition is privileged through embodied associations such as rightward/upward orientation, connection, and accumulation, whereas subtraction is linked to opposite mappings, including leftward/downward orientation, separation, and removal (Felisatti et al., 2021; Torbeyns et al., 2018; Lee and Schwarz, 2021). This cognitive imbalance is further reinforced by linguistic and cultural norms that equate “more” or “higher” with “better” (Glucksberg and McGlone, 2001) and tie accumulation to social status (Veblen, 1994). Taken together, these embodied, linguistic, and cultural dynamics help explain the systematic preference for additive over subtractive solutions.

Addressing additive bias is therefore critical across multiple levels of analysis: it can enhance individual cognitive flexibility and creative problem solving, strengthen organizational efficiency, and, at the broadest scale, promote more sustainable societal practices.

Overcoming this bias requires not only cognitive flexibility but also metacognitive awareness—the ability to reflect on one's own thinking and identify implicit assumptions (Flavell, 1979; Schraw and Dennison, 1994). While informing individuals about cognitive biases may seem like a straightforward solution, mere awareness is often insufficient to change behavior (Sheeran and Webb, 2016; Pronin et al., 2002)). More effective interventions are typically experiential, offering individuals direct engagement with tasks that challenge their default strategies and prompt reflective learning (Dewey, 1933; Schön, 2017; Weinstein, 1989; Ludolph and Schulz, 2018).

However, experiential learning requires time, resources, and carefully designed activities. In contrast, implicit measures such as the Implicit Association Test (IAT) offer a faster and more scalable means of revealing unconscious biases (Greenwald et al., 1998). Traditionally used to examine biases related to race, gender, and age, the IAT has been shown to prompt meaningful reflection and, in some cases, behavior change (Devine et al., 2012). Extending this methodology, a recent study by Neroni et al. (2024) introduced the additive bias IAT (ad-IAT), which measures individuals' implicit preference for additive over subtractive solutions. Their findings showed that the ad-IAT not only detects additive bias but also predicts behavioral tendencies in problem-solving contexts.

Building on this foundation, the present study investigates whether the ad-IAT can serve not just as a diagnostic instrument, but also as an educational tool, one capable of providing a rapid, personalized, and meaningful experience of one's own bias.

Given these insights, can we similarly develop and employ an IAT to uncover and educate people about the additive bias? A recent study by Neroni et al. (2024) has taken a step in this direction. Drawing inspiration from the IAT methodology, Neroni et al. developed the additive bias IAT (ad-IAT), a test designed to reveal individuals' implicit preference for additive over subtractive solutions, thereby capturing a cognitive bias that can shape real-world decision-making. Through four studies involving a large participant pool, Neroni et al. successfully demonstrated the ad-IAT's ability to identify and measure people's susceptibility to the additive bias and, notably, to predict future behavior. These findings underscore its ecological validity as a diagnostic tool and align with prior research showing that implicit measures can predict behavioral outcomes across problem-solving and decision-making contexts (e.g., Greenwald et al., 2009; Devine et al., 2012; Nosek et al., 2007).

Building upon this research, the study reported here aimed to explore the potential of the ad-IAT in educating individuals about the presence of the additive bias as a means of addressing it. Specifically, this preliminary investigation focused on whether the ad-IAT could be used to offer individuals an immediate, personalized, and meaningful experience of their own susceptibility to the additive bias. By providing this firsthand insight, the goal was to increase awareness of the additive bias and encourage individuals to counteract it by considering subtractive options.

The study consisted of three computer-based sessions. Session 1 involved familiarization with a digital grid task, which provided the foundation for the subsequent sessions. In Session 2, participants completed different tasks depending on condition: the ad-IAT group completed the ad-IAT (Neroni et al., 2024) and received personalized feedback; the Experiential Learning group engaged in a grid-based task designed to highlight the efficiency of subtractive strategies; and the Control group completed an unrelated gender IAT. In Session 3, all participants performed a grid-based task structured such that optimal performance required the use of a subtraction strategy. This design enabled an assessment of whether the interventions in Session 2 influenced participants' problem-solving approaches in Session 3. It was hypothesized that participants in the ad-IAT and Experiential Learning conditions would demonstrate greater openness to alternative strategies and a reduced reliance on additive solutions compared to those in the Control condition.

## 2 Method

### 2.1 Participants

Sixty participants (12 males) from Suor Orsola Benincasa University (Naples, Italy) were recruited through email lists and ads. Participants studied various fields, including psychology, education, law, and medicine, had an average age of 21.13 years (*SD* = 2.70) and were all native Italian speakers. One participant from the experimental group was excluded due to incomplete task performance, leaving a final sample of 59 participants. Before starting the study, participants read printed information sheets and gave their written consent to participate. Participants did not receive any honorarium in return for their participation. The entire study procedure was approved by Suor Orsola Benincasa University Ethics Committee.

### 2.2 Materials and procedure

At the beginning of the study, participants were randomly assigned to one of three experimental groups: ad-IAT, experience learning, and control. All participants were individually involved in three sessions: the task in Session 1 was common to all groups; the task in Session 2 varied according to the assigned condition; and the task in Session 3 was again the same for all groups. For all sessions and tasks, instructions were presented on a computer screen, and the study was conducted entirely via computer. All materials were administered in Italian^1^

An initial introduction explained to the participants that they would complete a series of computer-based tasks and answer some general questions about themselves. Then, participants were introduced to Session 1. In Session 1, participants were informed that they would familiarize themselves with a task they would complete later in the study. The task involved manipulating a digital grid pattern. Specifically, participants were given a 10 x 10 grid and were instructed to modify the colors of the grid cells. The grid was created and displayed through the Matlab software. Half of the grid blocks were green, while the remaining blocks were white, and participants could click on any square to observe how it changed color, transitioning between green and white or vice versa. They were allotted a 2-min window to interact with the grid before proceeding to Session 2.

For the ad-IAT group, Session 2 included the completion of the Italian version of the additive bias Implicit Association Test (ad-IAT, Neroni et al., 2024). The IAT was developed, hosted and administered through the Inquisit platform (https://www.millisecond.com/; Inquisit, 2024). During the ad-IAT, participants categorized stimuli as “pleasant” or “unpleasant” and “ADD” or “SUBTRACT” using different keyboard keys. The test included seven blocks. In the single categorization blocks (Blocks 1, 2, and 5), participants pressed a left key (“E”) if the stimulus belonged to the category presented on the left (e.g., “ADD, pleasant”) and the right key (“I”) for stimuli belonged to the category presented on the right (“SUBTRACT, unpleasant”). In the combined categorization blocks, the two stimuli sets were combined. In the combined compatible blocks (Blocks 3 and 4), participants pressed the left key (“E”) for “ADD” or “pleasant” and the right key (“I”) for “SUBTRACT” or “unpleasant.” Conversely, in the combined incompatible blocks (Blocks 6 and 7), participants pressed the left key (“E”) for “ADD” or “unpleasant” and the right key (“I”) for “SUBTRACT” or “pleasant.” For more details about the ad-IAT procedure see Neroni et al. (2024).

At the end of the ad-IAT, participants received personalized feedback on their performance. Following the conventional IAT procedure (for more examples see https://implicit.harvard.edu/implicit/takeatest.html), bias magnitude was classified as “slight,” “moderate,” or “strong” based on response times in various pairing conditions. For example, quicker responses for “ADD + Pleasant” compared to “SUBTRACT + Unpleasant” indicated a “strong automatic preference for adding concepts over subtracting concepts.” Participants also received a concrete behavioral example illustrating how their level of bias might manifest in problem-solving.

For the experiential learning group, Session 2 involved the completion of a modified version of the grids task originally devised by Adams et al. (2021). This task was created and presented using the Mathlab software. The task included three grids from Adams et al.'s study and two additional custom-designed grids. The use of five grids was intended to equalize the time needed to complete the task in Session 2, in alignment with the predetermined timeframes set in the other two experimental conditions. Participants were instructed to make each grid perfectly symmetrical using the minimum number of mouse clicks, a requirement explicitly emphasized by the bolded phrase “using the fewest possible mouse clicks.” All the five grids were set up so that achieving symmetry with additive transformations required more clicks than with subtractive transformations, thus requiring participants to consider subtractive actions to complete the task. Participants worked through the grids in a fixed order and did not receive feedback on their performance.

For the control group, Session 2 included the completion of an unrelated IAT, i.e., a gender IAT, typically used for the assessment of gender prejudice (e.g., Nosek et al., 2002). As for the ad-IAT, the IAT was developed, hosted and administered through the Inquisit platform. During the gender IAT, participants were asked to categorize attributes belonging to “Liberal Arts” vs. “Science” and target stimuli belonging to categories “Female” vs. “Male” into predetermined categories via keystroke presses. The remaining task procedure was identical to the ad-IAT. At the end of the task, feedback was provided similarly to the ad-IAT group.

**Figure 1:**
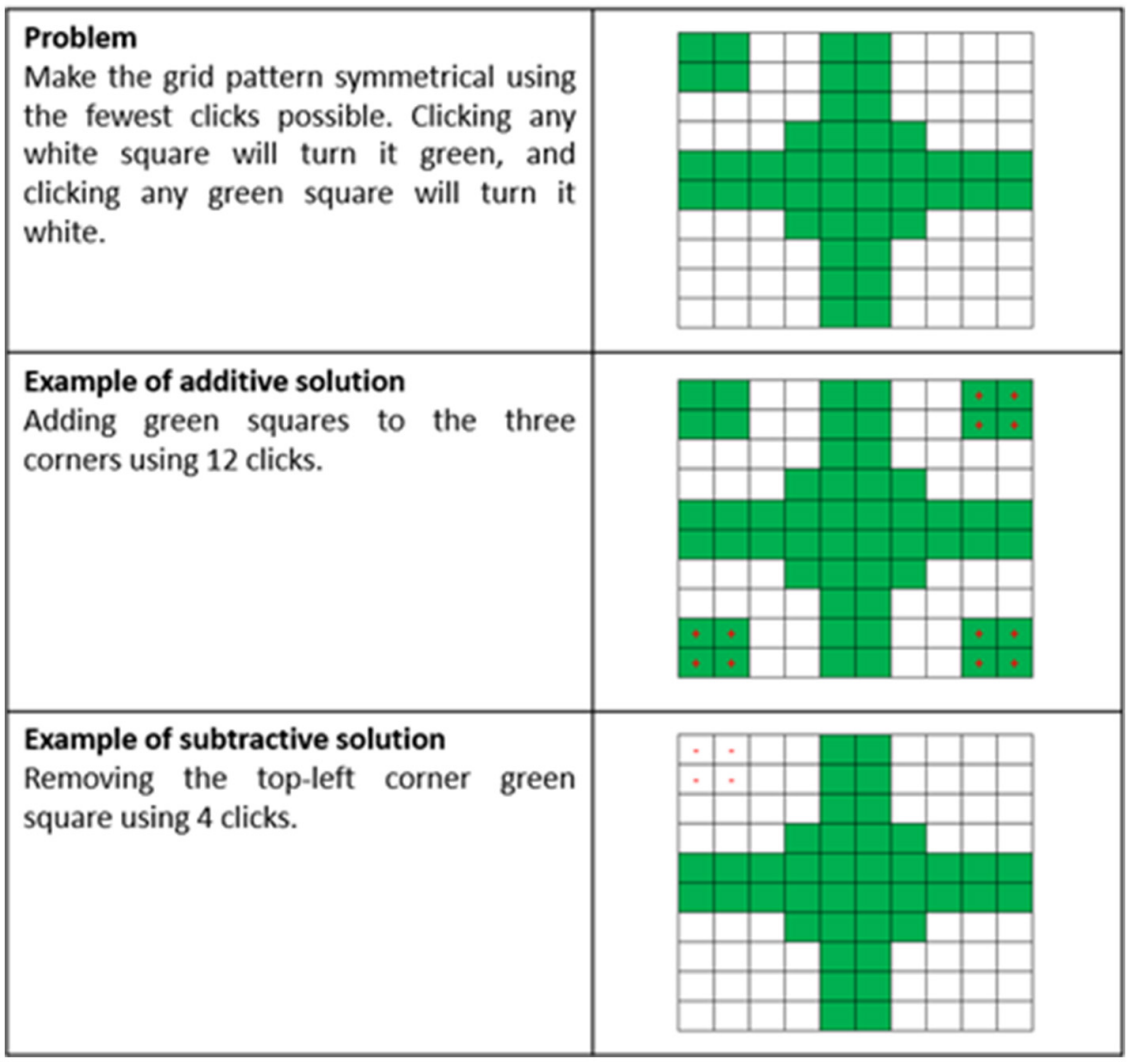
Grid used in Session 3 with description of additive and subtracting solution options.

After completing Session 2, all the participants moved to Session 3. For all groups, Session 3 included the completion a “test” grid taken from the Adams et al.'s (2021) study. The grid was developed and administered using the Mathlab software. This grid, similar to those used in Session 2 but with a different configuration, was designed such that achieving symmetry through additive transformations required more clicks than through subtractive transformations (see Figure 1).

Participants had unlimited time to complete the test grid. No feedback was provided during or after this task. At the end of Session 3, all the participants were asked to answer some demographic questions, indicating their sex, age, and field of study.

The total testing time was about 30 min per participant (see Table 1 for a summary of the activities completed by the participants in each group).

**Table 1:** Tasks and activities completed by the participants in each group.

**Session number**	**Experimental group**		
	**Ad-IAT**	**Experiential learning**	**Control**
Session 1	General instructions Grids task familiarization phase		
Session 2	Ad-IAT + individual feedback	Grids task practice	IAT gender + individual feedback
Session 3	Grids task: test grid Demographic questions		

## 3 Results

### 3.1 Baseline measure: additive bias IAT (session 2)

Participants in the ad-IAT group completed the additive bias Implicit Association Test (ad-IAT) during Session 2. The ad-IAT was scored using the D-score algorithm, which accounts for both the strength and direction of implicit associations (Greenwald et al., 2003). Positive D-scores reflected a stronger association between “additive” and “pleasant” stimuli (and “subtractive” and “unpleasant” stimuli), relative to the reverse pairings.

Participants in the ad-IAT group demonstrated a strong implicit preference for additive concepts. The mean D-score was 0.85 (*SD* = 0.38), indicating a pronounced bias associating “add” with “pleasant” and “subtract” with “unpleasant.” Reaction times were significantly faster in the compatible blocks (*M* = 948.65 ms, *SD* = 274.20) than in the incompatible blocks (*M* = 1,538.40 ms, *SD* = 336.57), *t*(19) = −8.79, *p* < 0.001, *d* = −2.02. Accuracy was also higher in the compatible blocks (*M* = 96.6%, *SD* = 3.3) compared to the incompatible blocks (*M* = 90.4%, *SD* = 8.9), *t*(19) = 3.78, *p* = 0.001, *d* = 0.87.

### 3.2 Behavioral measures: grid task (Session 3)

All participants completed the test grid task in Session 3. From this task, three dependent variables were derived:

Strategy type, coded by comparing the number of additive vs. subtractive clicks. Participants were classified as “additive” if additive clicks exceeded subtractive clicks, and “subtractive” otherwise. This allowed calculation of the proportion of participants who relied on the longer additive strategy within each condition.
Accuracy, coded dichotomously, with participants assigned a score of 1 if they achieved grid symmetry using the fewest possible clicks and 0 otherwise.
Task completion time, defined as the total latency (in milliseconds) from the onset of the grid until the final click that completed the symmetry transformation.

#### 3.2.1 Strategy use

Regarding the choice of additive strategies, the analyses revealed no significant group differences. The chi-square test was non-significant, χ^2^(2) = 1.30, *p* = 0.52, with a small effect size (Cramer's V = 0.15). Bayesian analyses converged with this conclusion, showing BF10 values around 0.2–0.3. However, descriptive trends suggested lower reliance on additive strategies in the experiential learning group (37%) compared to the ad-IAT (45%) and control (55%) groups.

#### 3.2.2 Accuracy

In terms of accuracy, results showed a trend toward group differences. Although the overall chi-square test did not reach the traditional threshold for statistical significance, χ^2^ (2) = 5.62, *p* = 0.060, Cramer's V = 0.31, descriptive statistics revealed that mean accuracy was higher in the experiential group (*M* = 0.47, *SD* = 0.51), followed closely by the ad-IAT group (*M* = 0.45, *SD* = 0.51), while the control group showed substantially lower accuracy (*M* = 0.15, *SD* = 0.37). The Bayesian analyses supported this pattern: the overall *BF10* was in the range of 2–3, suggesting anecdotal to moderate evidence for an effect of condition.

#### 3.2.3 Task completion time

For total task completion time, a significant effect of condition emerged, *F*_(2, 56)_ = 4.66, *p* = 0.013, η^2^ = 0.14. *Post-hoc* comparisons showed that participants in the experiential learning group completed the task significantly faster than both the ad-IAT group (*p* = 0.046) and the control group (*p* = 0.045), while the latter two did not differ from each other. Bayesian analyses provided strong support for this effect, with *BF10* values around 10–12 favoring the model including condition. Pairwise Bayes Factors confirmed moderate-to-strong evidence (BF > 6–8) that the experiential learning group outperformed the other two groups in terms of speed. *Post hoc* z-tests further indicated that the experiential learning group differed significantly from both the ad-IAT group (*z* = 2.02, *p* = 0.043) and the Control group (*z* = 2.24, *p* = 0.025), whereas the ad-IAT and Control groups did not differ (*z* = 0.36, *p* = 0.72).

## 4 Discussion

Additive bias refers to the tendency to solve problems by adding elements, even when subtraction would lead to more effective or elegant solutions (Adams et al., 2021). This cognitive default has implications not only for design inefficiencies and resource overuse (Frederiks et al., 2015; Page, 2009) but also for creative thinking, where it may constrain the generative space and suppress novel ideas (Crilly, 2015; Finke et al., 1992). While the concept of additive bias has recently gained empirical traction, strategies to mitigate its influence, particularly at an implicit level, remain underexplored. This pilot study examined the potential of the additive bias Implicit Association Test (ad-IAT) as a tool for increasing awareness of additive bias and encouraging more subtractive strategies in problem-solving. Drawing on prior research on cognitive biases and experiential learning (Crilly, 2015; Greenwald et al., 1998), the study sought to assess whether the ad-IAT, by surfacing implicit associations and providing personalized feedback, could promote metacognitive insight and behavioral change. It also compared this approach to a direct experiential learning condition, in which participants engaged with a task explicitly designed to highlight the efficiency of subtractive strategies.

Participants who completed the ad-IAT demonstrated a strong implicit preference for additive over subtractive concepts, as indicated by a significant IAT effect (*d* = 0.85). Although differences in behavioral strategy (additive vs. subtractive) were not statistically significant, participants in the ad-IAT condition were descriptively less likely than controls to default to additive solutions and showed greater task accuracy. These findings support the idea that the ad-IAT may serve as an effective reflective tool, helping individuals recognize implicit biases and shift cognitive tendencies through metacognitive awareness (Devine et al., 2012; Flavell, 1979).

The experiential learning group produced the most robust performance outcomes. Participants in this group completed the final grid significantly faster than both other groups and showed the lowest proportion of additive strategies. While no explicit feedback was provided, repeated exposure to grids in which subtractive transformations required fewer clicks than additive ones enabled participants to infer the greater efficiency of subtraction, fostering self-guided learning through experience. In this way, the task fostered self-guided learning through experience. These findings are consistent with the literature on “learning by doing” (Dewey, 1933; Schön, 2017; Weinstein, 1989), which highlights the power of embodied experience in reshaping cognitive patterns and improving decision-making.

These findings carry important implications for creativity research. Additive bias, by reinforcing default heuristics, undermines core aspects of creative cognition, including divergent thinking and problem reframing (Marsh et al., 1999; Ward et al., 1999). Interventions such as the ad-IAT may help make these constraints more salient, thereby fostering greater cognitive flexibility and originality. In educational contexts, for example, the ad-IAT could be incorporated into metacognitive training programs to encourage students to reflect on their problem-solving strategies, particularly during the early stages of ideation and problem framing (Getzels and Csikszentmihalyi, 1976; Veenman, 2013). Within design and innovation domains, it could also serve as a diagnostic tool for identifying shared cognitive defaults within teams, thereby enabling more diverse and generative idea development.

Beyond its applied value, the present study also contributes to theoretical accounts of additive bias by integrating insights from embodied and numerical cognition. Torbeyns et al. (2018) demonstrate that addition and subtraction are systematically shaped by embodied associations, with addition aligned to rightward and upward orientations and subtraction to leftward and downward ones. Lee and Schwarz (2021) extend this perspective with their concept of grounded procedures, showing that everyday physical actions such as cleansing, separating, or connecting can bias subsequent cognitive processing. Similarly, Felisatti et al. (2021) illustrate how separation and connection procedures map onto numerical concepts, linking embodied routines to abstract domains such as arithmetic. Taken together, these findings suggest that additive bias is not merely a limitation of abstract reasoning but is reinforced through embodied, linguistic, and cultural associations that privilege addition over subtraction. Within this framework, the present study advances the field by introducing the ad-IAT as a novel implicit measure that captures these deep-seated associations while also offering a practical tool with potential applications across education, design, and other domains where subtractive reasoning remains underutilized.

Despite the promising results, several limitations of this study must be acknowledged. First, the modest sample size constrained statistical power, rendering some effects only borderline significant. Given the sensitivity of *p*-values to sample size, such results should be interpreted cautiously. To address this issue, Bayesian analyses were also conducted, providing a more nuanced interpretation of the findings. Replication with larger and more diverse samples will be essential to establish the robustness and generalizability of these effects.

Second, methodological concerns raised in prior literature must be considered. Torbeyns et al. (2018) argue that task instructions may bias participants toward additive solutions, since terms such as *make* or *do* imply creation. Even the order of terms (“add and subtract” vs. “subtract and add”) can influence salience. In the present study, this confound was addressed by counterbalancing the order of terms across participants. Nonetheless, design features of grid-based tasks, such as the predominance of white over green squares, may still have rendered subtractive transformations less salient. Refinements in task design will be necessary to disentangle genuine cognitive tendencies from stimulus-driven biases.

Third, feedback provision varied across groups: participants in the ad-IAT condition received personalized results, those in the experiential learning condition received no direct feedback, and those in the control condition completed an unrelated IAT. This heterogeneity complicates the interpretation of findings and represents an important methodological limitation. Future studies should therefore more rigorously control for these differences by disentangling the influence of explicit feedback from the influence of the pre-test task itself, as both factors may have independently shaped participants' performance.

Finally, participants were not compensated, which may raise concerns about self-selection bias. Although participation was voluntary and compliance was high, future studies should consider incentivized participation to ensure more representative samples.

In conclusion, this study provides preliminary evidence that implicit bias tools such as the ad-IAT can increase self-awareness and reduce reliance on default additive strategies. When combined with experiential learning approaches, these tools may support more creative, efficient, and flexible problem-solving, particularly in contexts where subtractive reasoning is overlooked. By connecting additive bias to creativity as well as to embodied and numerical cognition, the present work underscores the value of integrating multiple theoretical perspectives to more fully understand and address this pervasive cognitive tendency.

## Data Availability

The raw data supporting the conclusions of this article will be made available by the authors, without undue reservation.

## Footnotes

^1^The full set of study materials is openly available on the Open Science Framework (OSF): https://osf.io/cfkje/?view_only=b6ca8b18120e461699ca4833c8d41ddf.
